# Genotypic and phenotypic properties of *Candida parapsilosis sensu strictu *strains isolated from different geographic regions and body sites

**DOI:** 10.1186/1471-2180-10-203

**Published:** 2010-07-28

**Authors:** Arianna Tavanti, Lambert AM Hensgens, Selene Mogavero, László Majoros, Sonia Senesi, Mario Campa

**Affiliations:** 1 Dipartimento di Biologia, Sezione di Genetica, Unità di Microbiologia, Università di Pisa, Via San Zeno 37, 56127 Pisa, Italy; 2 Dipartimento di Patologia Sperimentale, Biotecnologie Mediche, Infettivologia ed Epidemiologia, Università di Pisa, Via San Zeno 37, 56127 Pisa, Italy; 3 Department of Medical Microbiology, Medical and Health Science Center, University of Debrecen, Hungary

## Abstract

**Background:**

*Candida parapsilosis *is known to show limited genetic variability, despite different karyotypes and phenotypes have been described. To further investigate this aspect, a collection of 62 *sensu strictu C. parapsilosis *independent isolates from 4 geographic regions (Italy, n = 19; New Zealand, n = 15; Argentina, n = 14; and Hungary, n = 14) and different body sites (superficial and deep seated) were analysed for their genetic and phenotypic traits. Amplification fragment length polymorphism (AFLP) analysis was used to confirm species identification and to evaluate intraspecific genetic variability. Phenotypic characterisation included clinically relevant traits, such as drug susceptibility, in vitro biofilm formation and aspartyl protease secretion.

**Results:**

AFLP genotyping showed little variation among isolates, when the presence/absence of bands was considered. However, when AFLP profiles were compared by relative intensity for each fragment, a significant level of variation and geographical clustering was observed. All isolates were found to be susceptible to commonly used antifungals, although a reduced susceptibility to echinocandins was observed in all isolates. *C. parapsilosis *isolates from different geographic origins varied in the number of biofilm producers, with a higher prevalence of producers isolated in Hungary and Argentina. The frequency of secreted proteinase producers also varied in isolates obtained from different areas, with a higher number of proteinase producers found in Italy and New Zealand. Interestingly, biofilm production and proteinase secretion were negatively correlated. This finding could be explained by assuming that proteinase activity plays a role in detachment and release from a mature biofilm, via degradation of *C. parapsilosis *adhesins and/or extracellular matrix components, as observed for other microorganisms.

**Conclusions:**

The low number of polymorphic AFLP bands (18 out of 80) obtained for *C. parapsilosis *isolates is in agreement with the limited sequence variability described for this species. However, when band intensity was included in the analysis, geographical clustering was observed. Expression of virulence factors varied among strains isolated from different geographical regions, with biofilm and proteinase producers more frequently isolated from Hungary and Italy, respectively.

## Background

Of the species belonging to the "psilosis" group, *Candida parapsilosis *is by far the most studied and characterised. It represents about 90% of the infection attributed to *C. parapsilosis sensu lato *[1] and it seems to be better adapted to the human host than the two relatives (*C. orthopsilosis *and *C. metapsilosis*), as also shown by the high incidence of *C. parapsilosis *systemic infection worldwide, assessed as the second most common candidemia in many countries [2-6]. *C. parapsilosis *is an opportunistic pathogen that colonises human skin and can spread nosocomially through hand carriage [7,8]. It has been frequently associated with infections in newborns [6,8,9] and in catheterised patients [3]. This can be linked to the ability of *C. parapsilosis *to produce biofilm in the presence of plastic surfaces such as catheters or other prosthetic materials [6,10-12].

An increasing number of studies points towards a reduced genetic variability among *C. parapsilosis *isolates, which has been interpreted as a predominant clonal mode of reproduction [6,13-15]. This is in contrast to what has been recently described for *C. metapsilosis *and *C. orthopsilosis *species, in which recombination has been shown to occur by AFLP analysis [16,17]. On the other hand, a notable variability in virulence phenotypes has been observed for *C. parapsilosis*, such as the ability to produce biofilm or hydrolytic enzymes [6,18].

In this study, a selection of 62 *C. parapsilosis *isolates obtained from different patients was made from a wide collection of "psilosis" isolates on the basis of different geographical (Italy, Hungary, Argentina, New Zealand) and anatomical (blood, mucosa, nail, cerebrospinal fluid, etc) origins, in order to evaluate if these factors may have an impact on genetic variability.

AFLP was applied to our entire "psilosis" collection (n = 650), as this method has been shown to reproducibly and unequivocally identify Candida species [16,17,19]. The 62 selected isolates were analysed further by using another enzyme/primer combination EcoRI-HindIII, since the previously used EcoRI-MseI combination was found to be less discriminative and affected by band homoplasy in *C. parapsilosis *and *C. metapsilosis *[unpublished data, [17]]. The EcoRI/HindIII enzyme combination gives rise to larger fragments and therefore increases the sensitivity to detect polymorphisms.

In parallel, phenotypic properties such as biofilm formation and proteinase secretion were analysed. Since the "psilosis" species have been recently associated with a lower susceptibility to the echinocandin class of antifungals [20,21], drug susceptibility was also evaluated and extended to other antifungals. The overall goal of this study was to gain further information on genotypic and phenotypic properties of this successful and yet elusive opportunistic pathogen.

## Methods

### Isolates

The *Candida parapsilosis *collection included 62 individual isolates obtained from different body sites and geographical regions (Table 1). The majority of Italian isolates (n = 19) was provided by the Unità Operativa di Microbiologia, Ospedale Universitario, Pisa; 6 isolates being from different Italian hospitals (Table 1). Hungarian isolates (n = 14) were from the Department of Microbiology, Medical School, Debrecen. Argentinian and New Zealand isolates were kindly provided by Dr Marisa Biasoli, Centro de Referencia de Micologia, University of Rosario and by Dr Arlo Upton, Auckland City Hospital, respectively. The isolates used in this study were initially identified as *C. parapsilosis *according to their biochemical profile on API32 ID and a Vitek 2 advanced colorimetric semi automated system (bioMérieux, Marcy l'Etoile, France). *C. parapsilosis *ATCC 22019 was included in the study as reference strain. All isolates were maintained on Sabouraud agar (Liofilchem S.r.l., TE, Italy) for the duration of the study.

**Table 1:** Details and phenotypic properties of *Candida parapsilosis *clinical isolates used in this study.

Strain	Site of isolation	Origin	Biofilm^e ^30°C	Protease^f ^30°C
CP 1	Conjunctiva	Pisa (I)	0.006 (NP^i^)	0.3 (NP)
CP 17	Blood	Pisa (I)	0.015 (NP)	1.13 (WP)
CP 24	Blood	Pisa (I)	0.003 (NP)	3.0 (MP)
CP 28	Nail	Pisa (I)	0.006 (NP)	1.5 (WP)
CP 39	Blood	Pisa (I)	0.010 (NP)	1.0 (WP)
CP 42	Blood	Pisa (I)	0.042 (WP^l^)	0.5 (NP)
CP 66	Vaginal swab	Pisa (I)	0.001 (NP)	1.0 (WP)
CP 71	Vaginal swab	Pisa (I)	0.031 (WP)	1.0 (WP)
CP147^a^	Catether	Novara (I)	0.031 (WP)	0.3 (NP)
CP164^a^	Catether	Bergamo (I)	0.024 (NP)	3.5 (HP)
CP183^a^	Blood	Pavia (I)	0.012 (NP)	5.7 (HP)
CP 191^a^	Blood	Catania (I)	0.039 (WP)	1.25 (WP)
CP 192^a^	Blood	Catania (I)	0.034 (WP)	1.37 (WP)
CP 210^a^	Blood	Verona (I)	0.061 (WP)	2.0 (WP)
CP 243	Catether	Pisa (I)	0.019 (NP)	3.0 (MP)
CP 314	Sputum	Pisa (I)	0.017 (NP)	3.7 (HP)
CP 498	Vaginal swab	Pisa (I)	0.033 (WP)	1.9 (WP)
CP 499	Nail	Pisa (I)	0.019 (NP)	0.5 (NP)
CP 502	Oral swab	Pisa (I)	0.011 (NP)	4.2 (HP)
CP 425^b^	Blood	Auckland (NZ)	0.008 (NP)	4.0 (HP)
CP 426^b^	Blood	Auckland (NZ)	0.140 (MP^m^)	0.6 (NP)
CP 427^b^	Blood	Auckland (NZ)	0.040 (WP)	3.2 (HP)
CP 440^b^	Blood	Auckland (NZ)	0.060 (WP)	2.0 (WP)
CP 441^b^	Blood	Auckland (NZ)	0.031 (WP)	3.7 (HP)
CP 448^b^	Blood	Auckland (NZ)	0.127 (MP)	1.5 (WP)
CP 455^b^	Biopsy	Auckland (NZ)	0.416 (HP^n^)	0.2 (NP)
CP 459^b^	CAPD^g^	Auckland (NZ)	0.027 (NP)	2.2 (MP)
CP 471^b^	Vaginal swab	Auckland (NZ)	0.042 (WP)	1.0 (WP)
CP 476^b^	Vaginal swab	Auckland (NZ)	0.230 (HP)	0.7 (NP)
CP 477^b^	Vaginal swab	Auckland (NZ)	0.032 (WP)	2.8 (MP)
CP 479^b^	Nail	Auckland (NZ)	0.021 (NP)	2.25 (MP)
CP 480^b^	Nail	Auckland (NZ)	0.120 (MP)	1.2 (WP)
CP 481^b^	Nail	Auckland (NZ)	0.005 (NP)	3.0 (MP)
CP 486^b^	Urogenital swab	Auckland (NZ)	0.006 (NP)	2.0 (WP)
CP 540^c^	Faeces	Rosario (RA)	0.006 (NP)	2.5 (MP)
CP 541^c^	Urine	Rosario (RA)	0.015 (NP)	2.0 (WP)
CP 543^c^	Blood	Rosario (RA)	0.049 (WP)	0.5 (NP)
CP 544^c^	Blood	Rosario (RA)	0.111 (MP)	0.5 (NP)
CP 545^c^	Liquor	Rosario (RA)	0.046 (WP)	0.5 (NP)
CP 546^c^	Biopsy	Rosario (RA)	0.048 (WP)	1.3 (WP)
CP 550^c^	Liquor^h^	Rosario (RA)	0.100 (MP)	0.5 (NP)
CP 551^c^	Liquor	Rosario (RA)	0.058 (WP)	1.7 (WP)
CP 552^c^	Liquor	Rosario (RA)	0.047 (WP)	1.2 (WP)
CP 553^c^	Liquor	Rosario (RA)	0.033 (WP)	0.5 (NP)
CP 554^c^	Blood	Rosario (RA)	0.031 (WP)	1.5 (WP)
CP 555^c^	Blood	Rosario (RA)	0.101 (MP)	1.2 (WP)
CP 556^c^	Faeces	Rosario (RA)	0.078 (WP)	1.7 (WP)
CP 558^c^	Absess	Rosario (RA)	0.093 (MP)	1.0 (WP)
CP 510^d^	Blood	Debrecen (H)	0.083 (MP)	0.7 (NP)
CP 511^d^	Blood	Debrecen (H)	0.170 (HP)	0.1 (NP)
CP 512^d^	Catether	Debrecen (H)	0.167 (HP)	0.2 (NP)
CP 514^d^	Blood	Debrecen (H)	0.180 (HP)	0.5 (NP)
CP 521^d^	Urine	Debrecen (H)	0.058 (WP)	0.7 (NP)
CP 523^d^	Oral swab	Debrecen (H)	0.163 (PP)	0.5 (NP)
CP 524^d^	Ear swab	Debrecen (H)	0.049 (WP)	1.1 (WP)
CP 525^d^	Blood	Debrecen (H)	0.078 (WP)	1.0 (WP)
CP 527^d^	Blood	Debrecen (H)	0.032 (WP)	2.5 (MP)
CP 528^d^	Sputum	Debrecen (H)	0.009 (NP)	1.5 (WP)
CP 530^d^	Wound	Debrecen (H)	0.069 (WP)	1.1 (WP)
CP 531^d^	Urine	Debrecen (H)	0.037 (WP)	0.5 (NP)
CP 533^d^	Catether	Debrecen (H)	0.191 (HP)	0.4 (NP)
CP 536^d^	Catether	Debrecen (H)	0.162 (HP)	0.9 (NP)

^a^Strains CP147, 164, 183, 191, 192, 210 were kindly provided by Prof. G. Morace; ^b^New Zealand, ^c^Argentinean and ^d^Hungarian isolates were generously given by Dr Arlo Upton, Dr Marisa Biasoli and Dr Laszlo Majoros, respectively. ^e^Optical density (OD λ_450 nm_) of biofilm production. Biofilm was scored as absent when OD at 450 nm< 0.03. ^f^Radius of proteolysis. Proteinase activity was considered absent when no clarification of the agar around the colony was visible (radius of proteolysis ≤ 1 mm). ^g^CAPD stands for continuous ambulatory peritoneal dialysis. ^h^Liquor stands for cerebrospinal fluid. ^i^NP, non producer; ^l^WP, weak producer; ^m^MP, moderate producer; ^n^HP, high producer.

### DNA extraction and molecular typing of *Candida parapsilosis*

Genomic DNA was extracted from yeast samples grown in Sabouraud broth, (Liofilchem) as previously described [16]. DNA quantity and integrity was assessed by gel electrophoresis.

AFLP analysis was used to confirm species identification and to evaluate the genetic relatedness of *C. parapsilosis *isolates. AFLP was performed on 50 ng of genomic DNA as previously described [16]. The restriction-enzyme combination EcoRI/HindIII was used in the first restriction/ligation step. The concentration of the HindIII adaptor was equal to EcoRI (0.45 μM). Sequences of the adapters and pre-selective primers used for AFLP analysis were as already reported [17]. Pre-selective, selective amplifications and gel electrophoresis conditions were performed as previously described [16]. AFLP profiles, ranging from 100 to 700 bases, were exported as a TIFF file and analyzed with the TotalLab TL120 software package (Nonlinear Dynamics Ltd, UK) to evaluate genetic variability within the species. DNA bands obtained for each isolate were size-matched. AFLP bands were defined by time (Rf value) and by the surface of the fluorescent peak they form, as recently described [17]. Only bands which were at least 0.5% of the lane volume present in at least one of the isolates were included in the analysis. Bands were considered to be absent as the surface of the peak was less than 0.03% of the lane volume.

Dendrograms were built by the TL120 software using the unweighted-pair group method using arithmetic means (UPGMA). For each pair of isolates, a similarity index (S_AB_) was calculated, ranging between 0 (complete non-identity) and 1.0 (identity). The S_AB _between the patterns for every pair of isolates A and B was computed by the formula S_AB _= 2*E*/(2*E*+*a*+*b*), where *E *is the number of bands shared by both isolates A and B, *a *is the number of unique bands in the pattern for isolate A absent in the pattern for isolate B, and *b *is the number of unique bands for isolate B not present in isolate A.

Since *C. parapsilosis *isolates displayed very little polymorphic fragments, but showed a great variation in band intensity, the latter parameter was included in genotype analysis. Thus, the quantity of each AFLP fragment was normalised as a percentage of the total quantity of the AFLP fragments for a given isolate and defined as relative intensity. For each isolate pair, the Pearson's correlation of the relative intensities % of all fragments present in the two isolates was determined: a correlation index of 1 corresponded to a complete identical pattern.

A distance matrix was obtained by subtracting the correlation between two AFLP patterns from 1 (distance = 1-correlation). This distance matrix was imported into the Treefit program [22] and used to produce a UPGMA dendrogram, which was visualised with the Treeview program [23,24].

### Biofilm formation

Biofilm production by *C. parapsilosis *clinical isolates was evaluated as previously described [16,17]. One set of plates was incubated at 37°C and another at 30°C without agitation. After 24 h, plates were washed and the optical density was measured (OD at 450 nm). Biofilm production was considered as absent (no production; NP), when the OD at 450 nm was lower than 0.03, weak (WP, 0.03 ≤ OD < 0.08), moderate (MP, 0.08 ≤ OD < 0.16), or high (HP, OD ≥ 0. 16) [16].

### Proteinase secretion assay

Yeasts were pre-grown in YEPD liquid medium (2% glucose, 1% yeast extract and 2% bactopepton, Difco, Detroit, MI, USA). *C. parapsilosis *isolates were analyzed for secreted proteolytic activity on solid medium containing bovine serum albumin (BSA) as the sole nitrogen source. The inducing medium containing 1.17% yeast carbon base (Difco); 0.01% yeast extract (Biolife, Milan, Italy); 0.2% BSA (pH 5.0) (BDH, Poole, UK) was sterilised by filtration and added to a solution of autoclaved (2%) agar. The number of blastoconidia was microscopically determined and yeast suspensions were adjusted to 10^6^cells/ml. Ten μl of each yeast suspension was inoculated in duplicate onto BSA agar plates and incubated at 30°C for 7 days. Proteolysis was determined by amido black staining of the BSA present in the medium as described by Ruchel and colleagues [25]. Proteinase activity was considered to be absent when no clarification of the medium around the colony was visible (radius of proteolysis < 1 mm), weak when a clear zone was visible (1 ≤ radius < 2 mm), moderate when the clarification radius was comprised between 2 and 3 mm and high, when the proteolytic halo exceeded 3 mm in radius.

### Antifungal susceptibility

The colorimetric broth micro dilution method SensititreYeastOne^® ^(YO-9, Trek Diagnostic Systems Inc., Cleveland, USA) was used to evaluate *C. parapsilosis *susceptibility to amphotericin B, fluconazole, posaconazole, itraconazole, voriconazole, 5-flucytosine and the echinocandins (caspofungin, micafungin, anidulafungin) as previously described [17]. According to manufacture instructions, the positive growth well was examined after 24 hour incubation. If the well was red, endpoint for antifungal could be interpreted, otherwise plates were incubated for a further 24 hours.

Antifungal susceptibility interpretation criteria were according to the Clinical Laboratory Standards Institute (CLSI) M27-A3 and M27-S3 documents [26,27]. Briefly, caspofungin MIC ≤ 2 (μg/ml) susceptible (S) and > 2 (μg/ml) non susceptible; fluconazole MIC ≤ 8 (μg/ml) S, MIC between 16 and 32 (μg/ml) susceptible dose dependent (S-DD), MIC ≥ 64 (μg/ml) resistant (R); itraconazole MIC ≤ 0.125 (μg/ml) S, MIC between 0.25 and 0.5 (μg/ml) S-DD, MIC ≥ 1 (μg/ml) R; voriconazole MIC ≤ 1 (μg/ml) S, MIC = 2 (μg/ml) S-DD, MIC ≥ 4 (μg/ml) R; amphotericin B MIC ≤ 1 (μg/ml) S; 5-flucytosine MIC ≤ 4 (μg/ml) S, MIC between 8 and 16 (μg/ml) intermediate (I), MIC ≥ 32 (μg/ml) R [25,26]. For S-DD category, susceptibility is dependent on achieving the maximal possible blood level of the antifungal [26,27]. *C. parapsilosis *reference strain ATCC 22019 was used as control.

### Statistics

Statistical analysis was performed using Instat software (GraphPad, USA). One-way ANOVA followed by Post-hoc test (Bonferroni) was used to evaluate the level of statistical significance of clustering. The association between biofilm and proteinase production was determined by Pearson's correlation coefficient (r). Differences between proteinase/biofilm producers versus non producers were examined using Fisher's exact test. A *P *value < 0.05 was considered statistically significant.

## Results

### Molecular typing of *Candida parapsilosis *isolates

**Figure 1:**
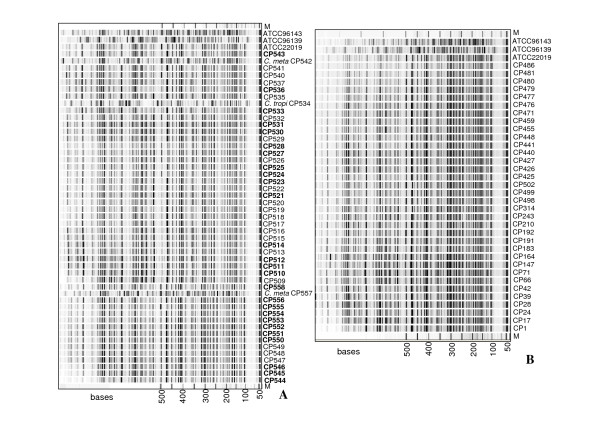
**AFLP patterns**. (A) AFLP profiles obtained from the molecular screening of 48 putative *Candida parapsilosis *clinical isolates and reference strains ATCC 22019 (*C. parapsilosis*), ATCC 96139 (*C. orthopsilosis*) and ATCC 96143 (*C. metapsilosis*). In bold, isolates used in this study for genotyping and phenotyping isolated from Argentina (CP540-558) and Hungary (510-536). M 50-500 base molecular weigh standard. In italics, the non-parapsilosis isolates identified during the AFLP screening. (B) AFLP profiles of 34 *C. parapsilosis *strains isolated from Italy (CP1-CP502) and New Zealand (CP425-486). At the top of the figure, reference strains for *C. metapsilosis *(ATCC 96143) *C. orthopsilosis *(ATCC 96139) and *C. parapsilosis *(ATCC 22019) are included.

AFLP was used to confirm correct species identification and to evaluate genetic variability within the selected 62 *C. parapsilosis *isolates. AFLP profiles obtained for *C. parapsilosis *consisted of 80 fragments ranging from 100 to 700 bases. Fragments larger than 700 bases were used as a control of DNA integrity. The number of monomorphic fragments was 62, which were common to all *C. parapsilosis *isolates. Therefore, these fragments were considered species specific and used for identification. Indeed, as shown in Figure 1A, which includes a wider panel of clinical isolates, this method allowed us to identify the presence of *C. metapsilosis *and *C. tropicalis *(CP542, CP534, CP557), which were excluded from this study. Identification of *C. tropicalis *and *C. metapsilosis *was performed by comparing AFLP profiles with those of 16 different fungal reference species [[16], data not shown].

**Figure 2:**
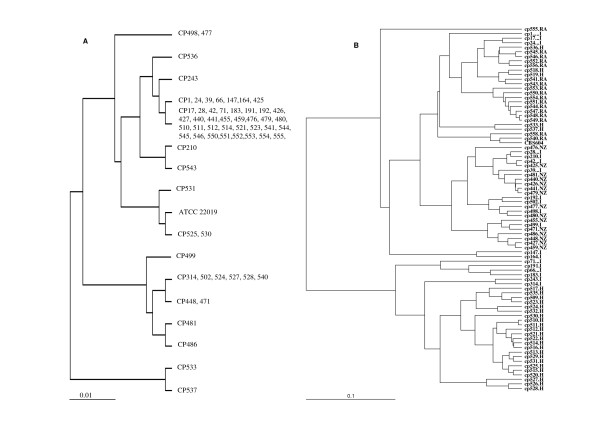
**AFLP profile analysis**. (A) UPGMA dendrogram based on presence/absence of AFLP fragments. (B) UPGMA dengrogram based on genetic distances derived from correlation coefficients including differences in relative band intensities. Geographical origin of isolates is indicated by I, Italy; RA, Argentina; NZ, New Zealand; and H, Hungary.

Figure 1 displays the AFLP profiles obtained from several *C. parapsilosis *isolates including those selected for the study and isolated from Argentina, Hungary (Figure 1A), Italy, and New Zealand (Figure 1B). When the presence/absence of fragments was the only parameter considered in AFLP analysis, very little genotypic diversity within the isolate collection was found (Figure 1A-B). In fact, the majority of AFLP markers included in the analysis (n = 80) were monomorphic, with only 18 polymorphic fragments. In agreement, UPGMA analysis indicated that all isolates grouped together, with a similarity index (S_AB_) higher than 0.96 (Figure 2A). A further level of analysis was performed to calculate a genetic distance between each isolate pair by the Pearson product-moment correlation coefficient (r), based on relative "intensity" of all fragments, which also takes into account the presence/absence of bands. Different band intensity was independent from DNA template concentration [data not shown; [28]], as expected, since AFLP is limited by primer concentration.

With this type of analysis, significant geographic clustering of the isolates was observed (Figure 2B). One-way ANOVA with Post-hoc test (Bonferroni) showed that all clustering above 0.04 (correlation coefficient of 0.96) were highly significant (*P *< 0.001). Reproducibility of the AFLP analysis was 97%, estimated by the average correlation among duplicated samples of reference *C. parapsilosis *strain (data not shown).

### Drug susceptibility

All *C. parapsilosis *isolates were found to be susceptible to the antifungals included in the SensititreYeastOne^® ^Y09 panel, with the exception of CP558 that displayed a dose-dependant susceptibility to fluconazole (MIC = 16 μg/ml). MIC values (μg/ml) were as follows: 5-flucytosine 0.06 ≤ MIC ≤ 0.25 (mean 0.127 ± 0.084 SD); posaconazole 0.008 ≤ MIC ≤ 0.5 (mean 0.069 ± 0.07 SD); voriconazole 0.008 ≤ MIC ≤ 0.5 (mean 0.037 ± 0.064 SD); itraconazole, 0.003 ≤ MIC ≤ 0.25 (mean 0.07 ± 0.036 SD); fluconazole, 0.125 ≤ MIC ≤ 16 (mean 1.8 ± 1.7 SD); amphotericin B, 0.125≤MIC≤ 1 (mean 0.44 ± 0.18 SD).

All *C. parapsilosis *isolates exhibited a reduced susceptibility to the echinocandin class, with the following MICs: anidulafungin, 0.5 ≤ MIC ≤ 2 (mean 1.32 ± 0.54 SD); micafungin, 0.5 ≤ MIC ≤ 2 (mean 1.17 ± 0.52 SD); caspofungin, 0.25 ≤ MIC ≤ 1 (mean 0.5 ± 0.22 SD).

All MIC values for echinocandin were ≤ 2 μg/ml (the defined cut-off value for susceptibility). However, caspofungin was the most active, with 85.5% of isolates showing MIC values ≤ 0.5 μg/ml.

### Biofilm formation

**Figure 3:**
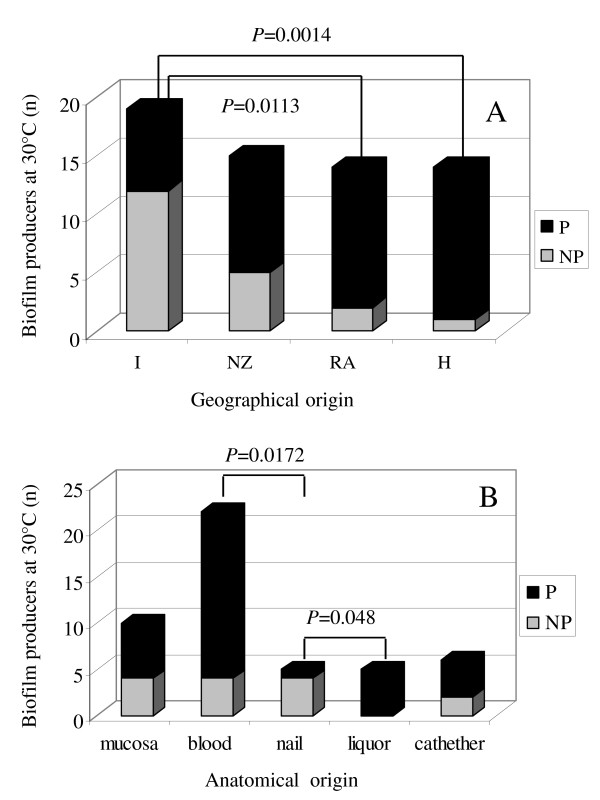
**Biofilm production by *C. parapsilosis***. Biofilm production following 24 h incubation at 30°C in inducing medium by *C. parapsilosis *isolates obtained from different geographical areas (A) and different anatomical sites (B). Liquor stands for cerebrospinal fluid. Number of biofilm producing isolates (P) versus non producers (NP) were compared using Fisher's exact test. A *P *value < 0.05 was considered statistically significant. I = Italy, NZ = New Zealand, RA = Argentina, H = Hungary.

To evaluate the effect of temperature on the formation of extra-cellular matrix, the production of biofilm was analyzed after 24 hour incubation at both 30°C and 37°C. As shown in Table 1, the majority of isolates produced biofilm (64.5%) following 24 hour incubation at 30°C, with similar results obtained after incubation at 37°C (64.3% of biofilm producers, data not shown). *C. parapsilosis *reference strain ATCC 22019 failed to produce biofilm at both temperatures tested. Statistically significant differences in the distribution of biofilm producers *vs *non producers were observed in strains isolated from different geographic regions. As shown in Figure 3A, the number of biofilm forming isolates (at 30°C) was significantly higher in isolates obtained from Hungary (*P *= 0.001) and Argentina (*P *= 0.011), compared with Italian strains, where a higher prevalence of non producers was found. The majority of isolates from New Zealand were biofilm producers. A similar trend was observed at 37°C (data not shown). When biofilm production was correlated with the anatomical origin of the samples, regardless of the geographical location, statistically significant differences in producers *vs *non producers could be observed between nail and blood isolates, with the latter encompassing a majority of biofilm producer strains, or between nail and cerebrospinal fluid samples (Figure 3B). Notably, all cerebrospinal fluid samples were isolated in Argentina. Again, results obtained at 30 and 37°C (data not shown) were similar. These experiments need to be confirmed with a wider range of isolates for each anatomical origin. Experimental variability was monitored by including a strong biofilm producer strain as a positive control in several experiments. Reproducibility experiments performed (n = 7) on separate days showed a mean absorbance of 0.348 ± 0.084 SD and a coefficient of variation of 24.1% [29]. The low standard deviation and a coefficient of variation of 24% indicated that good precision may be expected when using this method to estimate biofilm formation.

### Proteinase secretion

**Figure 4:**
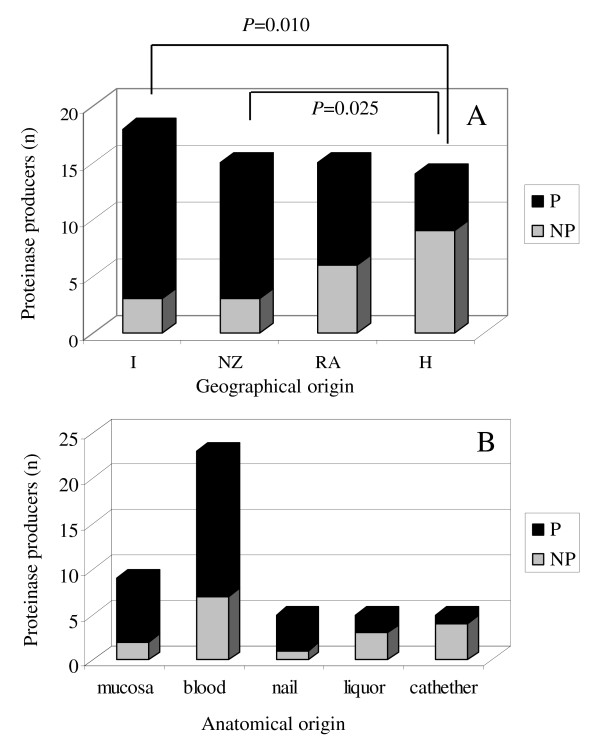
**Proteinase secretion by *C. parapsilosis***. Proteinase secretion by *C. parapsilosis *isolates obtained from different geographical areas (A) and different anatomical origin (B). 'Liquor' refers to cerebrospinal fluid. Proteolytic activity was assessed on YCB-BSA agar after 7 days incubation at 30°C. Numbers of protease producing isolates (P) versus non producers (NP) were compared using Fisher's exact test. A *P *value < 0.05 was considered statistically significant. I = Italy, NZ = New Zealand, RA = Argentina, H = Hungary.

Secretion of proteinase was measured as the proteolytic halo on solid BSA containing medium following 7 days incubation at 30°C. Most isolates were proteinase producers, with only 20 strains (33.9%) unable to hydrolyse BSA (Table 1). When the proteolytic activity was analysed in isolates obtained from different geographical regions an inverse trend was observed with respect to that obtained for biofilm production. In fact, a higher number of proteinase producers was found in Italy, and New Zealand, while they were significantly less represented in Hungary (*P *= 0.010 and 0.025, respectively, Figure 4A), where most biofilm producing strains were isolated. The analysis of protease production in isolates obtained from different body sites revealed no significant association between anatomical origin and production of this virulence factor (Figure 4B). The ATCC 22019 reference isolate showed no proteolytic activity (data not shown).

**Figure 5:**
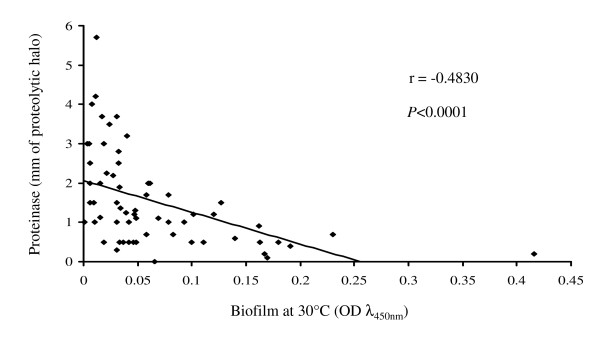
**Correlation between biofilm and proteinase production**. Negative correlation between biofilm production and proteinase secretion in *Candida parapsilosis *isolates (n = 62), as revealed by univariate regression analysis. Pearson's correlation coefficient (r) and *P*-value are indicated.

Univariate regression was applied to determine whether an association existed between the expression of the two virulence factors studied. As shown in Figure 5, a negative correlation between biofilm production and proteinase secretion by the *C. parapsilosis *isolates was observed (r = -0.483, *P *< 0.0001).

## Discussion

To date, no significant sequence variation has been described for *Candida parapsilosis *[30]. Therefore, this study was designed to provide further information on genotypic and phenotypic properties of this opportunistic fungal pathogen.

To evaluate the effect of different environments upon genetic variability *C. parapsilosis *isolates were selected to be representative of different geographical regions (Italy, Hungary, New Zealand, Argentina) and of different anatomical sites (blood, cerebrospinal fluid, mucosa, nail etc.). The EcoRI/HindIII enzyme combination used in the AFLP protocol was expected to produce a higher number of polymorphic bands since in *C. metapsilosis *band homoplasy was reduced with this combination and the average fragment length was larger than the one obtained with EcoRI/MseI [17]. Indeed the EcoRI/HindIII enzyme combination confirmed its higher discriminative power for *C. parapsilosis *and led to the identification of 20.7% of polymorphic fragments versus only 5% observed with EcoRI/MseI (data not shown). However, when genotype analysis was performed on the presence/absence of a band, the AFLP profiles obtained clearly indicated very high similarity, with all isolates grouped within a similarity index of 0.97.

This genetic variability is much lower than what we have observed for the species *C. metapsilosis *and *C. orthopsilosis*, for which we observed a greater number of polymorphic bands [16,17]. Our results are in agreement with the observation that the frequency of single nucleotide polymorphisms (SNPs) in *C. parapsilosis *is 30 to 70 fold lower than in other *Candida *species [30]. The low level of variability found suggests a clonal or selfing strategy of reproduction, supporting the hypothesis of a successful species recently emerged as a genetically homogeneous population diverged from a common ancestor [31]. It is not possible to determine whether the low variability found is due to a real absence of sexual recombination, as supported by the finding of a defective mating-like type locus [30,32], since the very limited number of variable fragments hinders a statistical analysis to prove/disprove the existence of recombination within this species, as previously performed for *C. orthopsilosis *and *C. metapsilosis *[16,17]. Interestingly, a recent manuscript by Sabino and colleagues [33] reports a high degree of polymorphisms by microsatellite analysis in *C. parapsilosis*, with 192 different genotypes found among 233 isolates, based on 4 hyper variable loci. This is remarkable, considering that the majority of the literature points towards limited genetic variability in this species. The hypervariability found can provide an excellent tool to discriminate between isolates in outbreak investigations. However, it does not seem to be useful for genetic relatedness studies on larger time scale or on population structure [33].

When the genetic distance between each isolate pair was calculated using the Pearson's coefficient, which takes into account both the presence/absence of bands and their relative "intensity", significant geographic clustering of the isolates was obtained (*P *< 0.001). This coefficient has been used as an index of genetic distance and has been previously reported in AFLP analysis of bacteria [34,35] and Candida species [36]. Candida fingerprinting techniques such as RFLP with species specific probes, RAPD, karyotyping also produce band patterns which differ in band mobility and intensity. In this respect, genotyping with AFLP gives rise to a much more complex pattern, composed by a larger number of bands, which can be compared by mobility and intensity [37]. The accuracy of the Pearson's coefficient is also dependent on the number of fragments included in the comparison. Thus, generating over 80 fragments with a single enzyme/primer combination, AFLP seems to be a suitable tool to perform this kind of analysis [37].

In this context, it is interesting to speculate what causes the variation in the relative band intensities. Karyotypes differing in band mobility and intensity have already been described for *C. parapsilosis *and other *Candida *species [[38], data not shown] and Butler and co-authors showed that *C. albicans *can be partially hemizygous [30]. The role that ploidy plays in *C. parapsilosis *genetic variability is a phenomenon already described. In fact, it was shown that its nuclear size ranges from 57% to 86% from its estimated diploid size [30,39]. We assume that one haploid complete set of the genome (50%) is always present in the isolates but what the remaining 7 to 36% of the DNA actually represents remains unknown. Whether this represents between 7 to 36% of one homologous set and/or whether these are DNA sequences present in variable copy numbers is still to be determined.

Using AFLP with the enzyme combinations EcoRI, HpaII, and MspI, we have noted that in *C. parapsilosis*, methylation of cytidine occurs. It was also observed that this methylation was variable in different isolates (data not shown). Variable DNA methylation of restriction sites might be responsible for different AFLP profiles, including the observed differences in relative intensities of AFLP bands found in this study with the enzymes HindIII and EcoRI.

For what concerns phenotypic traits, drug susceptibility tests showed that all isolates were susceptible to the antifungals tested, with the exception of one fluconazole dose-dependant susceptible isolate. Regardless of the geographical or anatomical origin, a reduced susceptibility to echinocandins was observed for all isolates, confirming what has already been described for this species [40]. It has been suggested that this phenotype is due to a naturally occurring Proline to Alanine amino acid change (P660A) in the glucan synthase enzyme Fks1p [40]. However, MIC values were all ≤ 2 mg/ml, the accepted breakpoint for echinocandins against *Candida *species [26,27].

Since this fungal pathogen is able to colonise body sites with different core temperatures, we examined whether biofilm formation was influenced by incubation at 30 or 37°C. The results obtained indicated that this parameter does not significantly alter the ability to produce biofilm in vitro, with minor differences in the quantity of the extracellular matrix produced at different temperatures. Interestingly, biofilm production was linked to both geographical and anatomical origin of isolates; indeed, Argentinian or Hungarian isolates produced significantly more biofilm than Italian strains. To date we do not have an explanation to justify the higher biofilm production that was observed in Hungarian isolates. The majority of these high biofilm producers came from surgery or intensive care units, where catheter related infections with biofilm producer isolates are more commonly found. Of note, even though the analysis was performed on a limited number of isolates, blood and cerebrospinal fluid isolates were found to be more frequently biofilm producers than strains isolated from nails. These findings need to be confirmed by comparing a wider set of isolates for each anatomical site of origin.

The majority of *C. parapsilosis *isolates (66.1%) produced proteinase in vitro. In contrast to what was observed for biofilm production, proteinase producers were mostly detected in Italy and New Zealand. Interestingly, a statistically significant inverse correlation was found between proteolytic activity and the ability to form biofilm, independent of the geographical/anatomical origin of isolates. Indeed, this finding has also been described for *Staphylococcus aureus *[41], where extracellular proteases make a significant contribution to a biofilm deficient phenotype of an *S. aureus *mutant, as shown by the addition of proteinase inhibitors to biofilm formation assay [41]. In addition, Boles and Horswill [42] demonstrated through genetic analysis that an *S. aureus *double mutant in a metalloprotease and serine protease, displaying minimal extracellular protease activity, was improved in biofilm formation, and had a strongly attenuated detachment phenotype. These findings demonstrate that the *S. aureus *dispersal mechanism from consolidated biofilm requires extracellular protease activity. Recently, the existence of a new pathway has been demonstrated, controlling protein-mediated biofilm formation in which different global regulators modulate biofilm formation by controlling the expression of *S. aureus *extracellular proteases [43]. Therefore, in analogy to what is described for *S. aureus*, we hypothesise that the negative impact of extracellular proteases on biofilm formation is multifactorial, potentially promoting detachment and release from a mature biofilm, via degradation of *C. parapsilosis *adhesins and/or extracellular matrix components.

## Conclusions

Overall, these results confirm previous evidence that *Candida parapsilosis *is characterised by a limited DNA sequence variability, even when considering isolates collected from distant geographical regions. The fact that phenotypic properties were found to significantly differ in strains isolated from various geographical regions suggests that other mechanisms such as epigenetic modifications may be used by this yeast to adapt to environmental changes.

## Authors' contributions

AT designed the study with LAMH, performed phenotypical analysis and drafted the manuscript; LAMH conceived the study with AT, performed AFLP analysis and wrote the manuscript; SM participated in the drug susceptibility assays; LM has made substantial contribution to acquisition of data and critically revised the manuscript. SS participated in the study coordination and has made substantive contribution to data analysis; MC participated in the study design and has given the final approval to the version to be published.

All authors have read and approved the final version of the manuscript.
